# Genome-wide identification and characterization of the SPL gene family and its expression in the various developmental stages and stress conditions in foxtail millet (*Setaria italica*)

**DOI:** 10.1186/s12864-022-08633-2

**Published:** 2022-05-20

**Authors:** Dili Lai, Yue Fan, Guoxing Xue, Ailing He, Hao Yang, Chunlin He, Yijing Li, Jingjun Ruan, Jun Yan, Jianping Cheng

**Affiliations:** 1 College of Agriculture, Guizhou University, Huaxi District, Guiyang, 550025 Guizhou Province People’s Republic of China; 2 School of Food and Biological Engineering, Chengdu University, Longquanyi District, Chengdu, 610106 Sichuan Province People’s Republic of China; 3 College of Food Science and Engineering, Xinjiang Institute of Technology, Aksu, 843100 People’s Republic of China; 4 College of Coastal Agricultural Sciences, Guangdong Ocean University, Zhanjiang, 524000 People’s Republic of China; 5 Henan Cancer Hospital, Zhengzhou, 450001 People’s Republic of China

**Keywords:** *Setaria italica*, SPL, Genome-wide, Abiotic stress

## Abstract

**Background:**

Among the major transcription factors, SPL plays a crucial role in plant growth, development, and stress response. Foxtail millet (*Setaria italica*), as a C4 crop, is rich in nutrients and is beneficial to human health. However, research on the foxtail millet *SPL* (SQUAMOSA PROMOTER BINDING-LIKE) gene family is limited.

**Results:**

In this study, a total of 18 *SPL* genes were identified for the comprehensive analysis of the whole genome of foxtail millet. These *SiSPL* genes were divided into seven subfamilies (I, II, III, V, VI, VII, and VIII) according to the classification of the *Arabidopsis thaliana* SPL gene family. Structural analysis of the *SiSPL* genes showed that the number of introns in subfamilies I and II were much larger than others, and the promoter regions of *SiSPL* genes were rich in different cis-acting elements. Among the 18 *SiSPL* genes, nine genes had putative binding sites with foxtail millet miR156. No tandem duplication events were found between the *SiSPL* genes, but four pairs of segmental duplications were detected. The *SiSPL* genes expression were detected in different tissues, which was generally highly expressed in seeds development process, especially *SiSPL6* and *SiSPL16,* which deserve further study. The results of the expression levels of *SiSPL* genes under eight types of abiotic stresses showed that many stress responsive genes, especially *SiSPL9*, *SiSPL10*, and *SiSPL16*, were highly expressed under multiple stresses, which deserves further attention.

**Conclusions:**

In this research, 18 *SPL* genes were identified in foxtail millet, and their phylogenetic relationships, gene structural features, duplication events, gene expression and potential roles in foxtail millet development were studied. The findings provide a new perspective for the mining of the excellent *SiSPL* gene and the molecular breeding of foxtail millet.

**Supplementary Information:**

The online version contains supplementary material available at 10.1186/s12864-022-08633-2.

## Introduction

Foxtail millet (*Setaria italica*) was first domesticated from the ancestor green foxtail around 16 000 Years Before Present (YBP) [1–3] and became the main crop in Northern China around 5000–6000 YBP [4–6]. As a diploid C4 crop, it has a great significance for energy utilization, with their photosynthesis especially improving nitrogen and water utilization efficiency [7, 8]. In addition, foxtail millet is self-compatible and has a highly conserved genome structure, which makes forward or reverse genetics and trait location easier [9]. Foxtail millet is rich in various nutrients, including phenolic compounds (phenolic acids, flavonoids, and tannins) [10], organic acids (gallic acid, caffeic acid, and chlorogenic acid) [11], lipophilic oxidants (vitamin E and carotenoids) [12], proline-rich prolamin (prolamin) [13] and trace elements (zinc and iron) [14]. As a model plant, foxtail millet is of great value in many aspects [2, 8, 15], but foxtail millet does not seem to have received enough attention at present. It is worth noting that, as a nutritionally balanced crop, it plays an important role in maintaining human health. Under the Covid-19 pandemic, studies have shown that foxtail millet has the potential to become a new staple food crop, especially in hunger hotspots [16]. Therefore, the study of foxtail millet is of both biological and socio-economic significance.

Transcription factors play an important role in plant growth and development. The SQUAMOSA promoter binding-like (SPL) protein family is a plant-specific transcription factor. The *SPL* genes encoded a highly conserved 76 amino acid long DNA-binding domain, namely the SBP domain [17–19]. The SBP domain has three important functional motifs, which are two zinc binding sites, namely Zn-1 (Cys-Cys-Cys-His) and Zn-2 (Cys-Cys-His-Cys) [20, 21], and a nuclear localization signal (NLS) located at the C-terminal [19]. In previous studies, SPL members were divided into eight groups, namely I-VIII [18, 22, 23]. The whole genomes of an increasing number of species have been sequenced, making it easy to identify gene families. In fact, many gene families in foxtail millet have been fully identified and analyzed, including bHLH [24], NAC [25], GRAS [26] and AP2/ERF [27]. At present, the SPL gene families of several species have been extensively studied, including dicotyledons (*Arabidopsis* [18, 28], tomato [22], Tartary buckwheat [23], grape [29], and cotton [30]), monocotyledons (rice [31, 32], wheat [33], and barley [34]), and C4 crops (maize and *Sorghum bicolor* [35]).

SPL is a key regulator of a variety of biological processes in plants, including changes from nutrition to the reproductive phase, leaf development, tiller/branching, plastid pigment, spike/spike structures, grain ripening, fertility, and response to stress [36]. In *A. thaliana*, *AtSPL2*, *AtSPL9*, *AtSPL10*, *AtSPL11*, *AtSPL13*, and *AtSPL15* play important roles in the transition from young plants to mature plants and from vegetative growth to reproductive growth. *AtSPL3*, *AtSPL4*, and *AtSPL5* play important roles in promoting the transformation of floral meristems [28]. *AtSPL14* can resist fumonisin B1 [37], and *AtSPL8* affects gibberellic acid biosynthesis and ultimately regulates reproductive development [38, 39]. There are also studies on the SPL gene in rice, such as *OsSPL14*, which regulates plant structure by inhibiting the number of tillers in rice, increasing grain weight and enhancing disease resistance [40–42]. *OsSPL16* promotes grain filling and improves the quality and yield of rice [43, 44]. In addition, *OsSPL3* can improve the cold resistance of plants [45]. Some of the wheat SPL genes have been found to be down regulated under NaCl and PEG treatments [33], while 13 *ZmSPL*s in maize were found to be involved in the drought stress response [35]. Since the SPL transcription factor family was found to be targeted by miR156 [18, 46], the gene function of SPL has been more widely studied. The regulatory pathway of the miR156/SPL module is highly conserved in different plant species and plays an important role in regulating plant fitness, biomass, and yield [36]. Moreover, as plants age, the level of miR156 decreases, thus alleviating the inhibition of SPL targets [47–49].

SPL plays a crucial role in plant growth, development, and stress response. Overall, the identification, classification, evolution, and gene function studies of *SPL* gene families in foxtail millet are not systematic. Therefore, this study comprehensively analyzed the sequence composition, gene structures, cis-acting elements, miR56 binding sites, chromosome positions, and gene replication events of 18 SPL gene families in the millet genome. The evolutionary relationship of *SiSPL* genes were also analyzed among several species, including rice, *A. thaliana*, sorghum, maize, tomato, and tartary buckwheat. The grouping, motif composition, collinearity, and evolutionary relationship between the *SiSPL* genes and other plants were analyzed. We also studied the spatial expression and tissue expression patterns of *SPL* genes in different tissues during foxtail millet development. As a result, the roles of specific SPL gene members in different biological processes of foxtail millet were determined. In addition, the expression of *SPL* genes in foxtail millet under eight types of abiotic stress were investigated. In this study, a comprehensive analysis of the SPL gene family of foxtail millet was carried out, which not only screened important *SPL* genes under growth, development, and stress treatment, but also provided insights for the study of the SPL gene family of other plants.

## Results

### Identification of SPL proteins in foxtail millet

We used two blast methods to extract *SPL* genes from the whole genome of foxtail millet. After removing redundant genes, a total of 18 *SPL* genes were obtained. These 18 *SPL* genes were mapped to the nine chromosomes and renamed *SiSPL1* to *SiSPL18* according to their location. The characteristics of these *SiSPL* genes, including coding sequence length, amino acid sequence length, molecular weight of protein, isoelectric point of protein, and prediction of protein subcellular localization, were analyzed. Among the 18 *SPL* genes, the smallest protein had only 181 amino acids (SiSPL4), while the largest protein had 1118 amino acids (SiSPL14). The molecular weight of the protein ranged from 19.2 kDa to 122.18 kDa with an isoelectric point ranging from 5.58 to 9.91. The results of subcellular localization showed that a total of 16 genes were located in the nucleus, one gene was localized in the plastid, and the other was located in the chloroplast (Additional file Table S1).

### Multiple sequence alignment, phylogenetic analysis, and classification of *SiSPL* genes

**Fig. 1:**
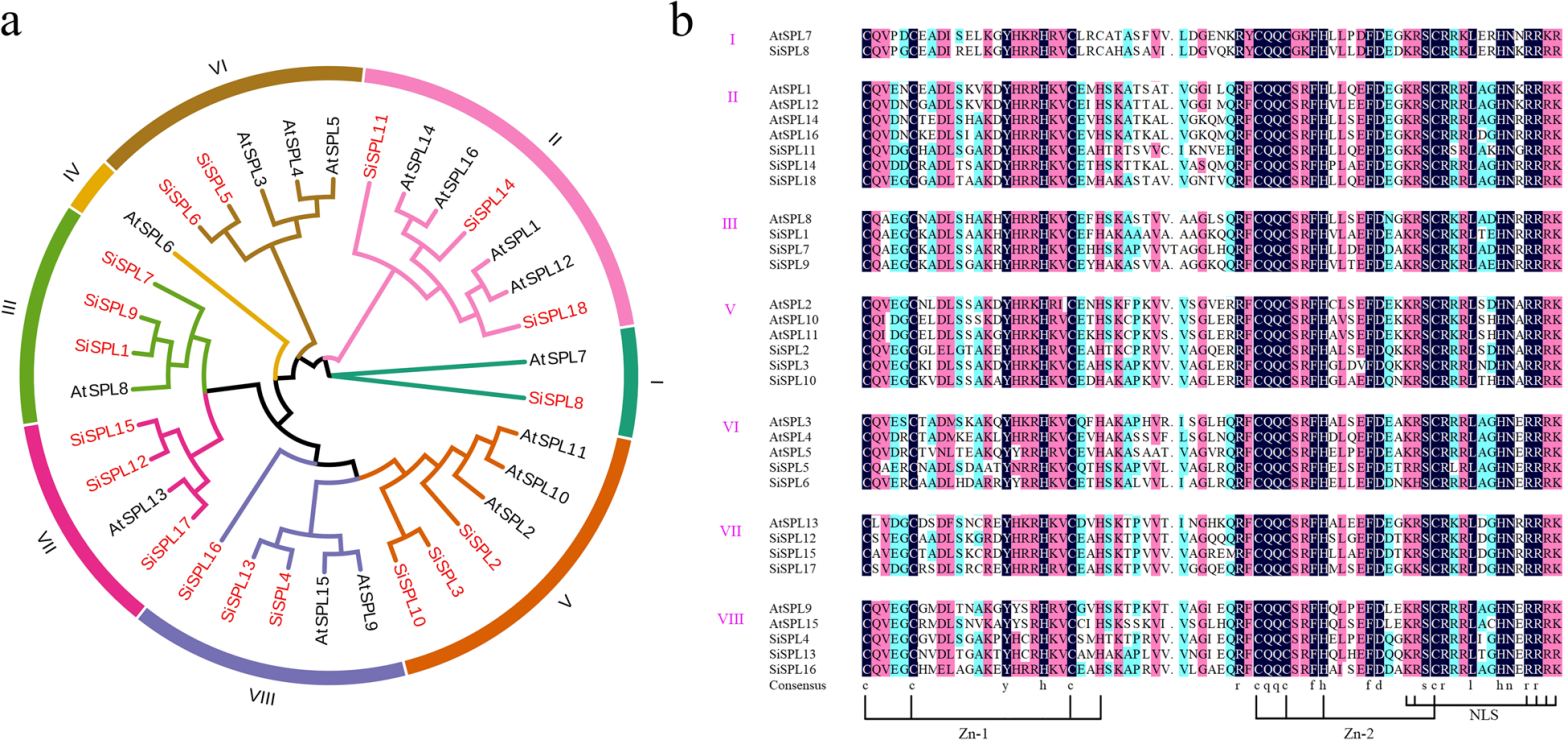
Unrooted phylogenetic tree of the relationship between *Setaria setaria* and *Arabidopsis thaliana* SPL proteins, and SBP domain sequence alignment. The phylogenetic tree was derived using the ML method in MEGA X. The tree shows the 8 phylogenetic subfamilies. **a** Phylogenetic tree of the relationship between *S. setaria* and *A. thaliana* SPL protein. **b** 76 bp sequence alignment of SBP domain

To explore the evolutionary relationship of *SPL* genes in foxtail millet, 16 *A. thaliana SPL* genes and 18 *SiSPL* genes were used to construct a phylogenetic tree using MEGAX software. According to the classification method of the *AtSPL* gene family, as shown in the evolutionary tree, the SPL gene family can be divided into eight subfamilies, namely groups I to VIII (Fig. 1a). However, there were no *SiSPL* members in group IV, and the final *SiSPL* gene family was divided into seven subfamilies. Among these subfamilies, group I contained the least *SiSPL* gene members, only one (*SiSPL8*), followed by group VI with two members (*SiSPL5* and *SiSPL6*), while the other subfamilies contained three *SiSPL* members. According to the location of the SBP domain, we extracted the SBP domain sequence of the *AtSPL* and *SiSPL* genes (approximately 76 amino acids) and used them for multiple sequence alignment. As shown in Fig. 1b, the foxtail millet SBP domain contains highly conserved sequences, such as CQQC, SCR, and RRR. In addition, a highly conservative nuclear localization signal (NLS) was found in the foxtail millet domain. Two zinc finger-like structures, Zn-1 and Zn-2, were also found in the SBP domain. There was a certain mutation in the Zn-1 (Cys-Cys-Cys-His) sequence of subfamily I, and the fourth histidine was mutated to cysteine. Therefore, the conformation of zinc finger binding site 1 of subfamily I may be mutated, conferring a special function to subfamily I. However, this phenomenon was not observed in other subfamilies containing highly conserved Zn-1 (Cys-Cys-Cys-His) and Zn-2 (Cys-Cys-His-Cys).

### Gene structure and motif composition of the SiSPL gene family

**Fig. 2:**
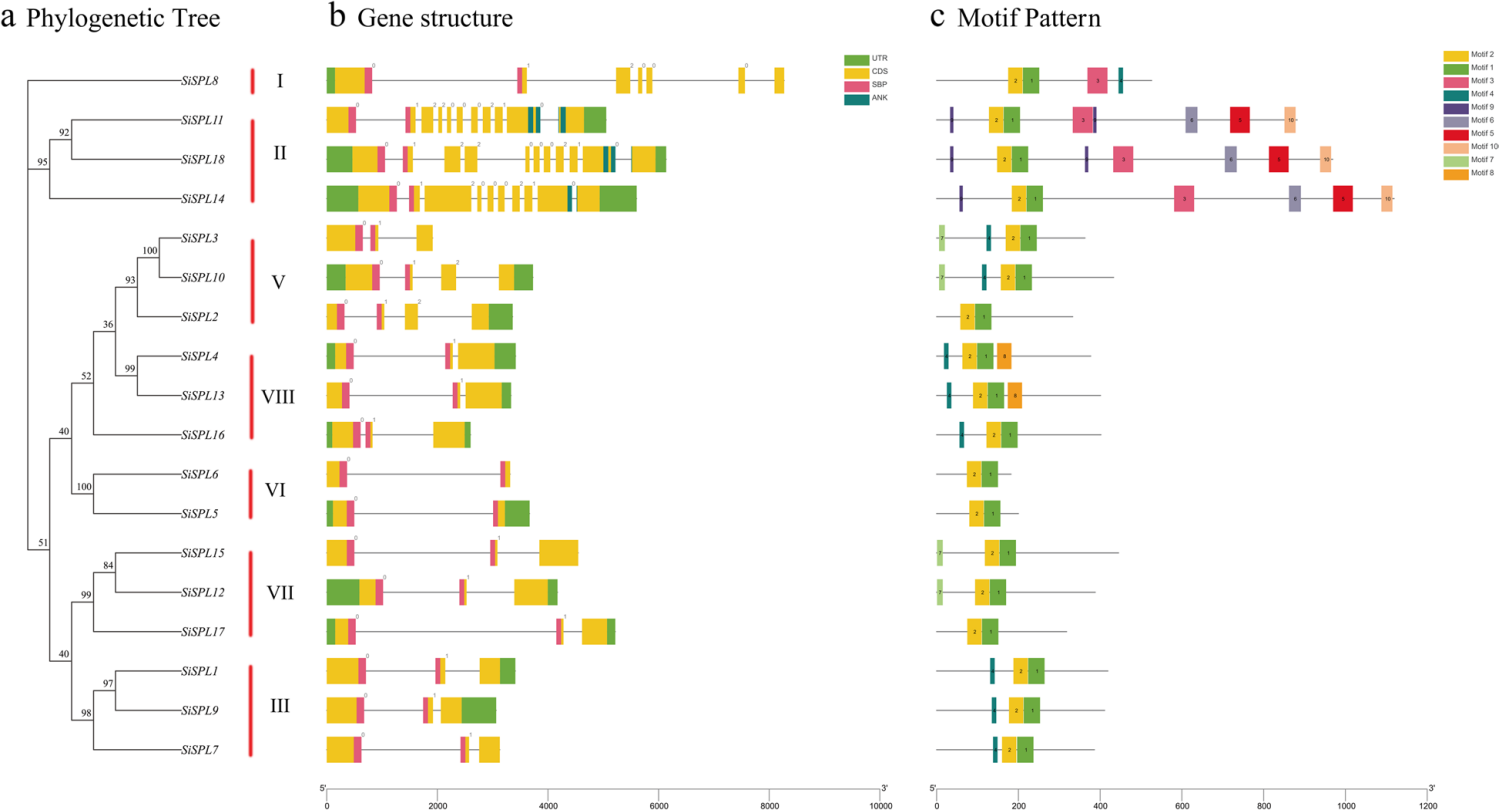
Phylogenetic relationships, gene structure and architecture of the conserved protein motifs in 18 genes from *S. italica*. **a** The phylogenetic tree was constructed based on the full-length sequences of *Si*SPL proteins. **b** Exon–intron structure of *Si*SPL genes. Lines represent introns, boxes represent exons, and domains are color-coded. Number indicates the phase of the corresponding intron. **c** Amino acid motifs in the *Si*SPL proteins (1–10) are represented by colored boxes. Black lines indicate relative protein lengths. Sequence information for each motif is provided in Additional file Table S2

The evolution of the SPL gene family in foxtail millet was further explored by studying the intron–exon structures of all *SiSPL* genes. As shown in Fig. 2, while the intron–exon structures of the same subfamily were similar, while the differences of different subfamilies were large. For example, subfamily II (*SiSPL11*, *SiSPL14*, and *SiSPL18*) had the largest number of introns, with an average intron number as high as 9.7. Second, *SiSPL8* (subfamily I) contained six introns, while the other subfamilies had a small number of introns, with an average of only two introns. Subfamily VI, *SiSPL5* and *SiSPL6*, which have the least number of introns, only had one intron. All SPL members have an SBP domain, and interestingly all SBP domains are separated by introns. In addition members of subfamily III also possess ANK domain.

To explore the differences in the conserved motifs of each foxtail millet SPL protein, we used the MEME website to analyze the motif composition of the entire sequence, including the sequence of the SBP domain. Ten different conserved motifs were identified from these sequences, namely motifs 1 to 10 (Fig. 2c, Additional file Table S2). Motifs 1 and 2 were distributed among all *SiSPL* members, motifs 9/6/5/10 were unique to subfamily II (*SiSPL11*, *SiSPL14*, and *SiSPL18*), and none of the other subfamilies contained these four motifs. In addition, subfamily VIII (*SiSPL4* and *SiSPL13*) also contained a special motif, motif 8. The motif arrangement of the same subfamily was similar, indicating that these protein structures were conserved relatively, which supported the reliability of the *SiSPL* subfamily classification.

### Cis‑acting element for the *SiSPL* gene family

The promoter region 2-kb upstream of the *SiSPL* genes were further analyzed. A total of 86 cis-acting elements were found in the promoter region of these *SiSPL* genes (Additional file Table S3). They were divided into seven categories, including promoter-related elements, light-response elements, hormone response elements, environmental stress-related elements, development-related elements, binding site-related elements, and other elements. Among these elements, light reaction-related elements (20) and development-related elements (14) accounted for a larger proportion. Light reaction-related elements (G-box, Sp1, and TCCC-motif) are present in most *SiSPL* genes. Development-related elements, such as root-specific expression (as-1) and meristem expression element (CAT-box), are also widely distributed in the *SiSPL* gene family. In addition, we also found that most *SiSPL* genes had cis-acting elements involved in abscisic acid (ABRE), methyl jasmonate (TGACG-motif, CGTCA-motif), and auxin (TGA-element), while a few genes also had cis-acting elements involved in gibberellin (P-box, GAREmotif), ethylene (ERE), and salicylic acid (TCA-element). Among the environmental stress-related elements, hypoxia inducible (GC motif), low temperature response (LTR), and drought inducible-related elements (MBS) were found to be widely distributed in *SiSPL* genes. Among the promoter-associated elements, the core elements associated with transcription initiation (TATA and CAAT box) were present in all *SiSPL* genes, which proves that our promoter analysis is reliable. Finally, 25 other cis-acting elements were found in foxtail millet *SPL* genes, of which STRE (AGGGG) and Unnamed_4 (CTCC) were found in all *SiSPL* genes; however, the functions of these elements are unknown.

**Fig. 3:**
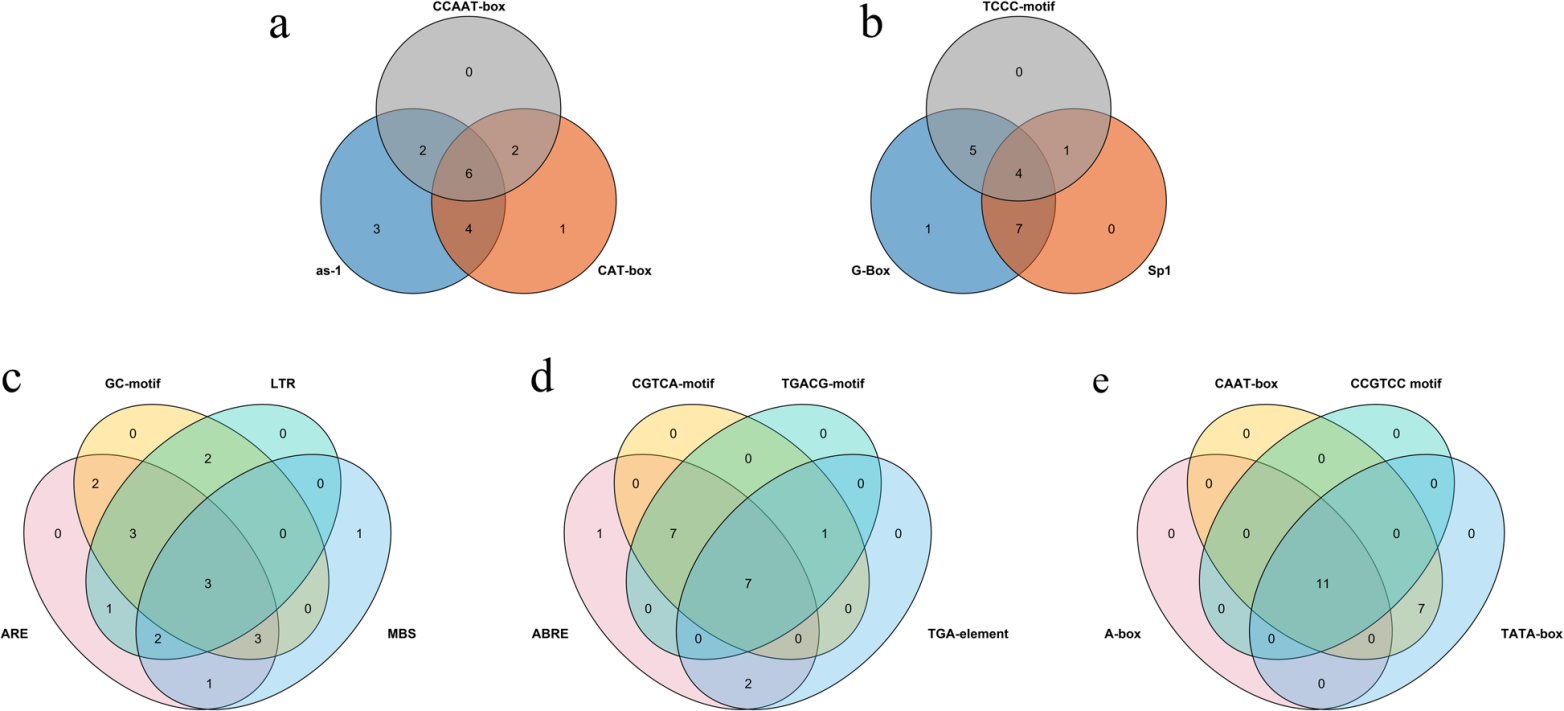
Promoter cis-elements of **a** development-related elements, **b** light-responsive elements, **c** environmental stress-related elements, **d** hormone-responsive elements, and **e** promoter-related elements

In addition, Venn analysis was performed on cis-acting elements contained in more than 10 genes (Fig. 3) to further analyze the significance of these elements. For example, among the environmental stress-related elements, the three *SiSPL* genes (*SiSPL9*, *SiSPL16*, and *SiSPL2*) contained ARE (AAACCA), GC-motif (CCCCCG), LTR (CCGAAA), and MBS (CAACTG) cis-acting elements. Among the light response-related elements, four *SiSPL* genes (*SiSPL10*, *SiSPL15*, *SiSPL14*, and *SiSPL4*) contained G-Box, Sp1, and TCCC-motif elements at the same time. Six *Si*SPL genes (*SiSPL11*, *SiSPL15*, *SiSPL16*, *SiSPL3*, *SiSPL4*, and *SiSPL8*) in development-related elements contained as-1, CA T-box, and CCAAT-box at the same time. Seven *SiSPL* genes (*SiSPL13*, *SiSPL15*, *SiSPL14*, *SiSPL1*, *SiSPL18*, *SiSPL2*, and *SiSPL4*) contained ABRE, TGACG-motif, CGTCA-motif, and TGA-element in the hormone response element.

### MiR156 family in foxtail millet and their target site in *SiSPL* genes

**Fig. 4:**
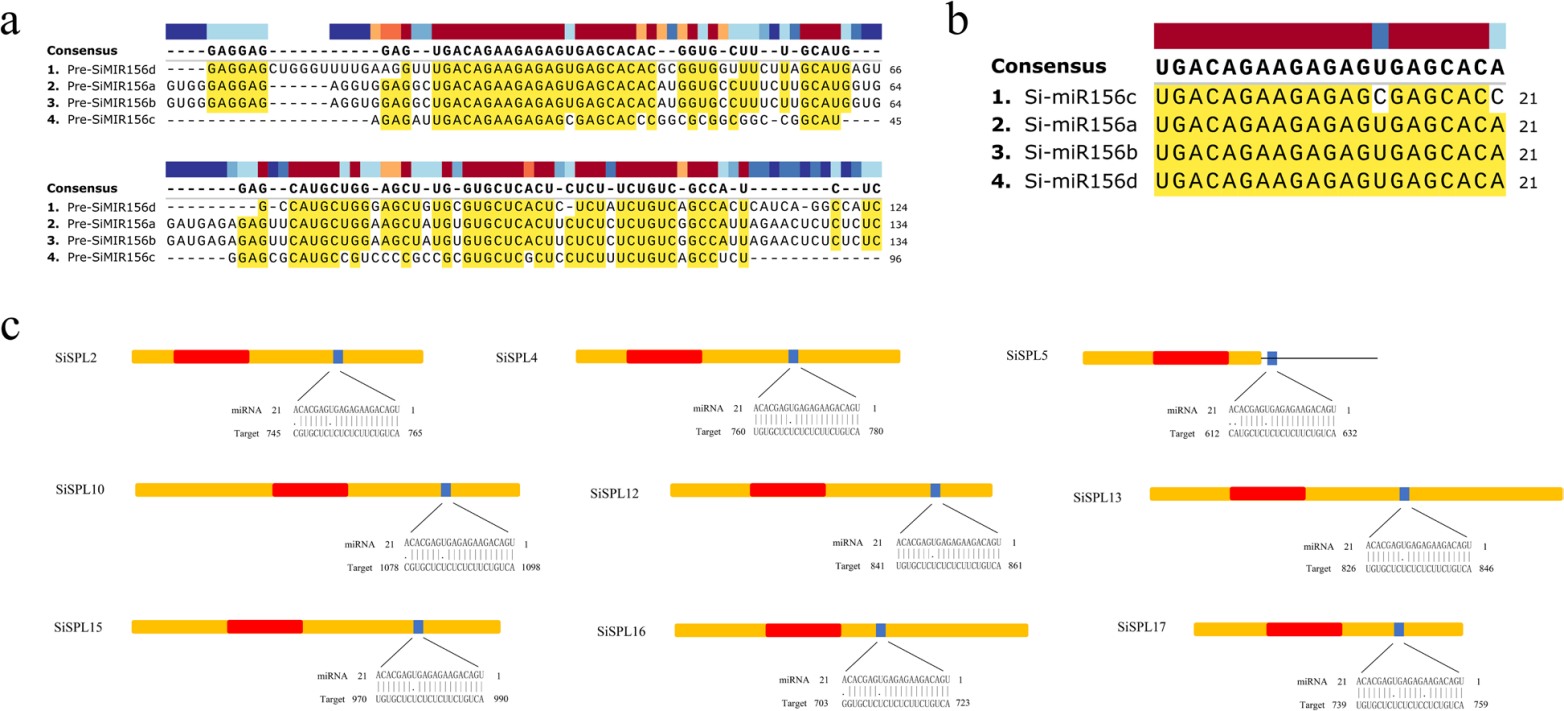
miR156 family members and their target site in *S. italica* SPL genes. **a** Alignment of precursor sequences of four miR156 family members. **b** Alignment of mature sequences of four miR156 family members. **c** miR156 target site in *Si*SPL2, 4, 5, 10, 12, 13, 15, 16 & 17 genes. Yellow box represent CDS, red box SBP domain and line 3′UTR. The miR156 target sites with the nucleotide positions of *Si*SPL transcripts are shown in blue. RNA sequence of each complementary site from 5’ to 3’ and the predicted miRNA sequence from 3’ to 5’ are indicated

In order to understand the post-transcriptional regulation of *SiSPL* mediated by miR156, we searched all *SiSPL* coding regions and 3’-UTR to find the target sites for miR156 to bind *SiSPL*. To this end, we studied the miR156 family in the whole millet genome and further analyzed their targets on these *SiSPL* genes. First, we downloaded the foxtail millet miR156 family from PMRD (http://bioinformatics.cau.edu.cn/PMRD/) and found four members of the miR156 family: miR156a, miR156b, miR156c, and miR156d (Fig. 4a, b). The sequences of mature miR156a, miR156b, and miR156d were identical, except for the fact that the sequence of mature miR156c differed by two bases. However, the precursor sequences of the four members differed considerably. We found a total of nine *SiSPL* with possible complementary binding to miR156 among the 18 genes. miR156-targeted *SiSPL* genes were distributed across groups V-VIII, with potential sites of binding to miR156 found in the coding regions of eight genes, namely *SiSPL2*, *SiSPL4*, *SiSPL10*, *SiSPL12*, *SiSPL13*, *SiSPL15*, *SiSPL16*, and *SiSPL17* (Fig. 4c, Additional file Table S4). The 3’-UTR region of *SiSPL5* identified a potential site for binding to miR156. These results indicate that miR156 may specifically regulate the expression of these *SiSPL* genes in foxtail millet. miR156 complementary sites are relatively conserved among these *SiSPL* genes, and the sequence differences are mainly limited to the first, second, and eighth nucleotides of the complementary sequence. This means that these complementary sites may be under great selection pressure in the process of evolution, even those located in the 3’-UTR. These results suggest that miR156-mediated post-transcriptional regulation plays an important role in the function of the foxtail millet *SPL* gene.

### Chromosomal distribution, gene duplication, and synteny analysis of the *SiSPL* genes

**Fig. 5:**
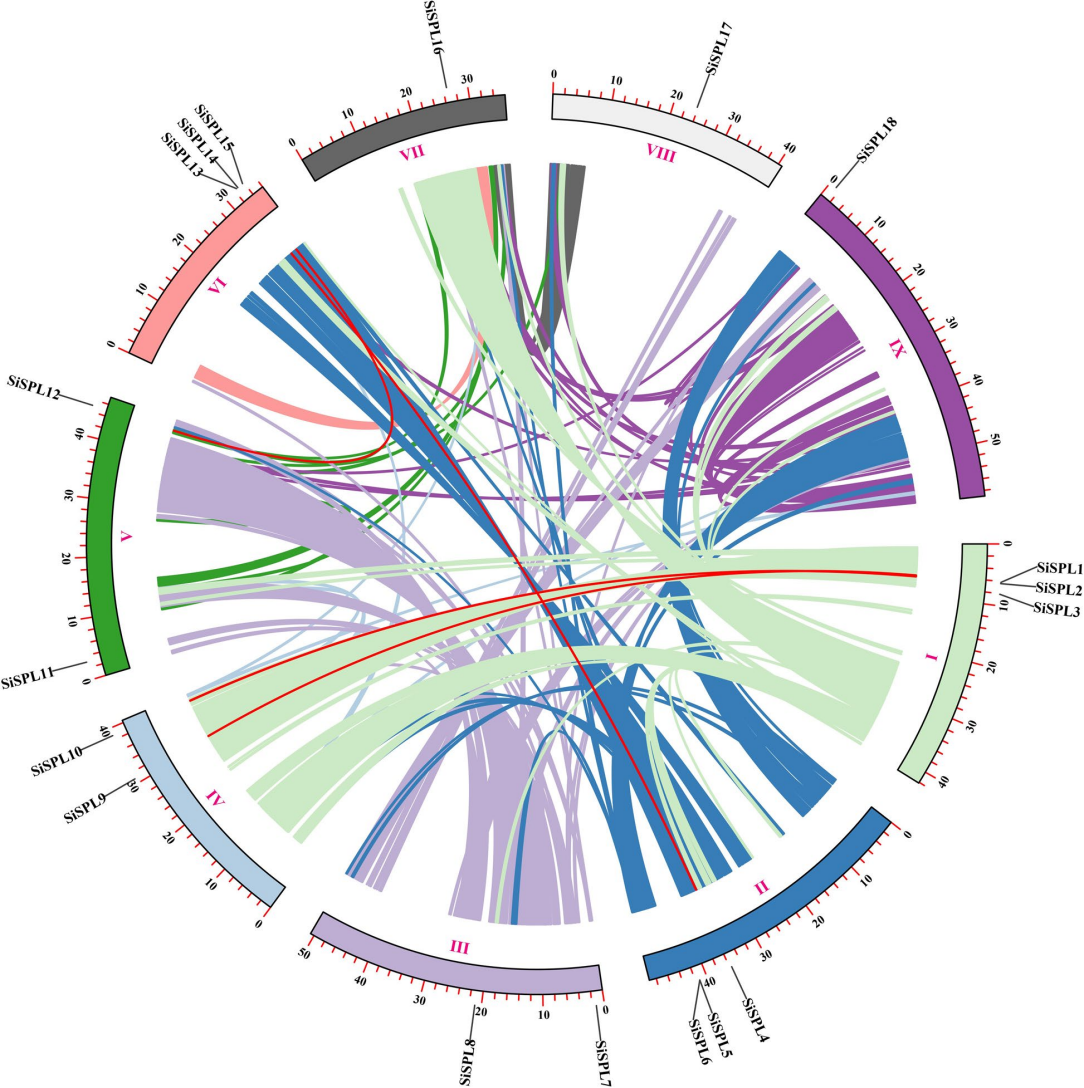
The chromosomal distribution and synteny blocks of the *S. italica* SPL genes. Schematic representation of the chromosomal distribution and interchromosomal relationships of *S. italica* SPL genes. Colored lines indicate all synteny blocks in the *S. italica* genome and red lines indicate segmental duplicated SPL gene pairs. Chromosome number is indicated at the bottom of each chromosome

The chromosomal location of 18 *SiSPL* genes was determined using the foxtail millet genome, which distributed in nine chromosomes of foxtail millet. The least number of *SiSPL* genes were distributed in chromosome VII, chromosome VIII, and chromosome IX with only one *SiSPL* gene each. The remaining chromosomes contained two or three *SiSPL* genes. Gene duplication events are essential for the evolution of gene families and tend to play an important role in gene expansion and the generation of new functional genes. Therefore, we investigated the duplication events of the *SiSPL* gene in the whole foxtail millet genome and found that there was no tandem repeat event for the *SiSPL* gene. However, four pairs of segmental duplication events were found in the SiSPL gene family (Fig. 5, Additional file Table S5) using the BLASTP and MCScanX methods. These segmental duplication genes are distributed on chromosomes 1/2/4/5/6, with each pair of segmental duplication genes is contained in the same subfamily. These results indicate that segmental duplication events play a major role in the expansion of the *SiSPL* gene in the evolution of the SiSPL gene family.

**Fig. 6:**
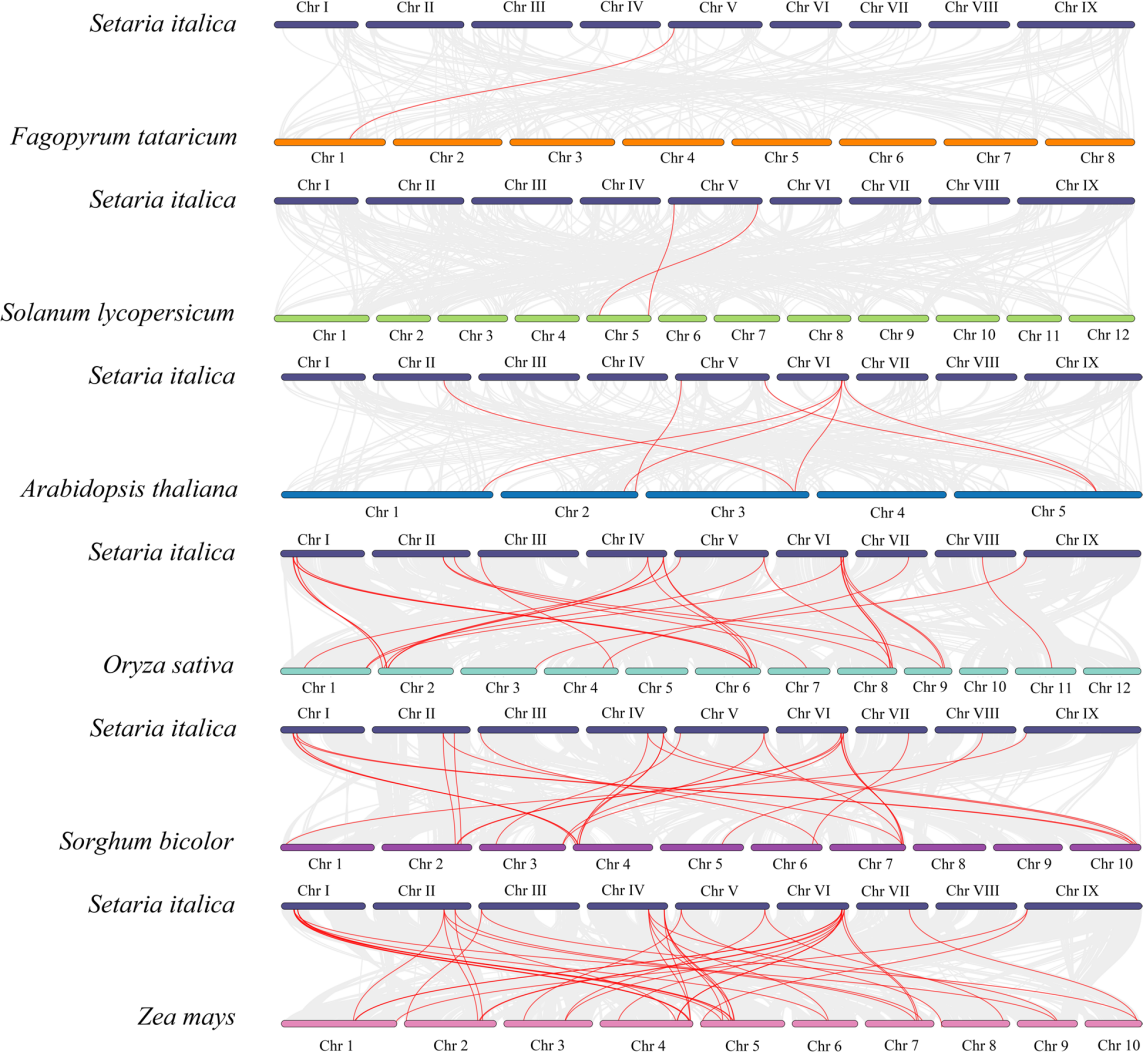
Synteny analysis of SPL genes between *S. italica* and other plant species. Synteny analysis of the SPL genes between *S. italica* and six representative plant species (*A. thaliana*, *F. tataricum*, *S. lycopersicum*, *S. bicolor*, *O. sativa*, *Z. mays*). Gray lines in the background indicate the collinear blocks within *S. italica* and other plant genomes, while red lines highlight the syntenic SPL gene pairs

To further study the phylogenetic mechanism of the foxtail millet SPL gene family, we constructed syntenic maps between six representative species with foxtail millet, which are three monocotyledons (rice, sorghum, and maize) and three dicotyledons (*A. thaliana*, tartary buckwheat, and tomato) (Fig. 6). Among the homologous genes of the foxtail millet *SPL* gene and other species, the homologous genes with maize was the most (42 pairs). This was followed by rice (28 pairs), sorghum (26 pairs), *Arabidopsis* (7 pairs), tomato (2 pairs), and tartary buckwheat with the least homologous genes (one pair). Interestingly, *SiSPL11* had homologous genes in all six species (Additional file Table S6). *SiSPL12* has homologous genes with five other species except tartary buckwheat, suggesting that *SiSPL11* and *SiSPL12* may have existed before the differentiation of monocotyledons and dicotyledons, and play important roles in these species after differentiation. In general, the foxtail millet *SPL* genes have the best homology with maize, and these highly homologous genes may have evolved from the common ancestor of different plants. To better understand the evolutionary constraints acting on the SiSPL gene family, the Ka/Ks values of the *SiSPL* gene pairs were calculated (Additional file Table S7). The results showed that the Ka/Ks values of the gene pairs of each subfamily and the gene pairs of all segmental repeats were all less than 1, suggesting that the SPL gene family of foxtai millet may have undergone strong purification selection pressure during evolution.

### Evolutionary analysis of SiSPL proteins and the *SPL* genes of several other species

**Fig. 7:**
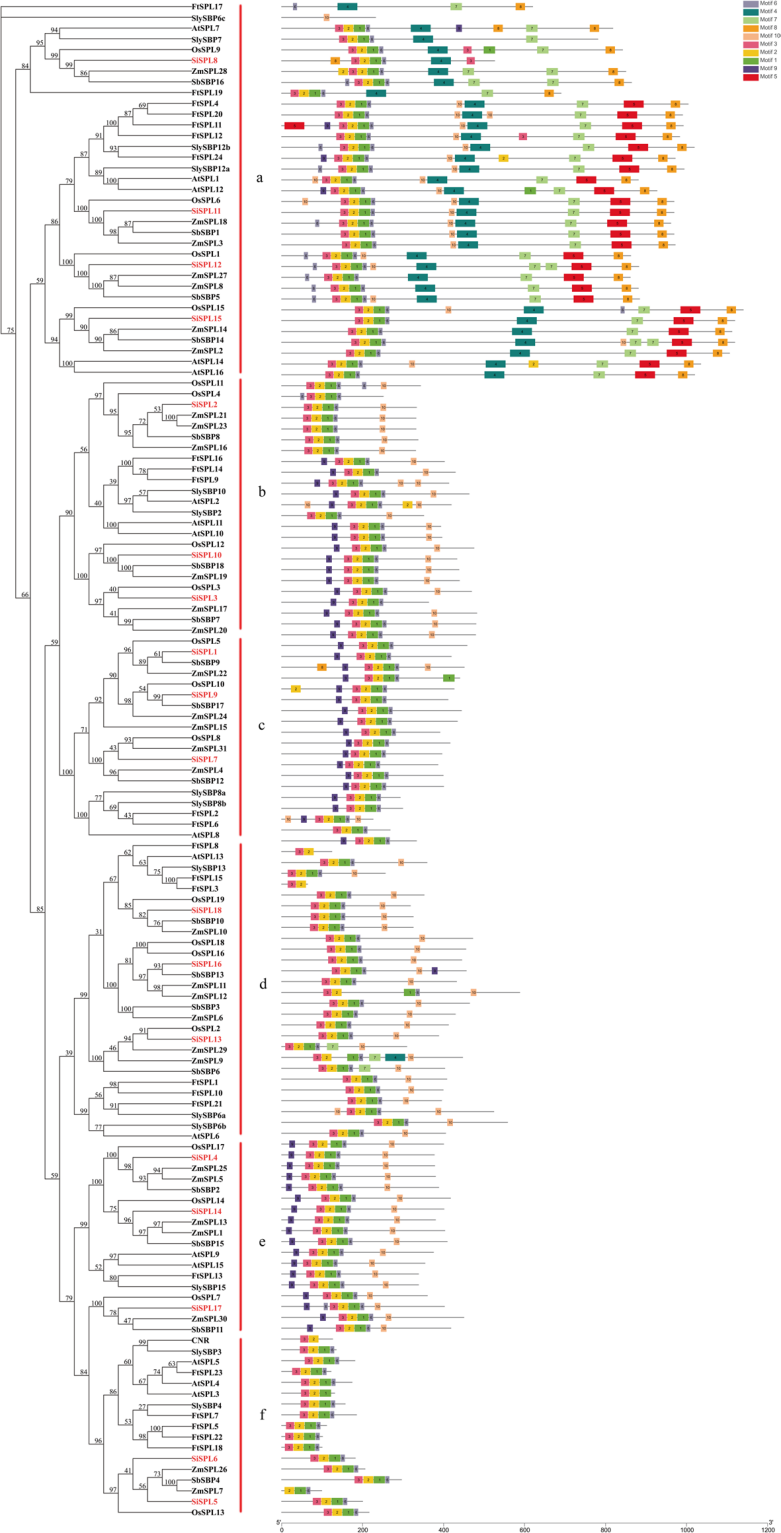
Phylogenetic relationship and motif composition of SPL proteins from *S. italica* with six different plant species (*A. thaliana*, *F. tataricum*, *S. lycopersicum*, *S. bicolor*, *O. sativa*, *Z. mays*). Evolutionary relationship between SPL proteins and motif composition. Different-colored boxes represent different motifs and their positions in each SPLprotein sequence. Sequence information for each motif is provided in Additional file Table S2

We constructed phylogenetic trees of SiSPL proteins with SPL proteins from monocots and dicots using Mega X. Among them, monocots included rice (19 proteins), sorghum (18 proteins), maize (31 proteins), and dicots including *A. thaliana* (16 proteins), Tartary buckwheat (24 proteins), and tomato (15 proteins) (Fig. 7, Additional file Table S8). As shown in Fig. 7, SPL proteins can be divided into six subfamilies in the phylogenetic tree (labeled a-f). Each of the seven species contributed to at least one *SPL* gene in different subfamilies, and 18 *SiSPL* genes were almost evenly distributed in these six subfamilies.

The conserved motifs of the SiSPL protein were analyzed using online MEME analysis. The motifs 3, 2, 1, and 6 were conserved and distributed almost alternately across the whole subfamily. This indicates that this motif composition is very important for these *SPL* genes and may be of great significance for the SPL protein. However, the differences between different subfamilies were also relatively large. This included subfamily a that contained conserved motifs 4/7/5/8, while the remaining subfamilies did not contain. Subfamilies b and e contained special motifs 9 and 10. Subfamily c contained special motif 9 but lacked motif 10. The situation in subfamily d was the opposite to that in subfamily c. Subfamily f showed the most differences, containing motifs 3, 2, 1, and 6, and no other conserved motifs.

### Expression patterns of the *SiSPL* genes in different foxtail millet tissues

**Fig. 8:**
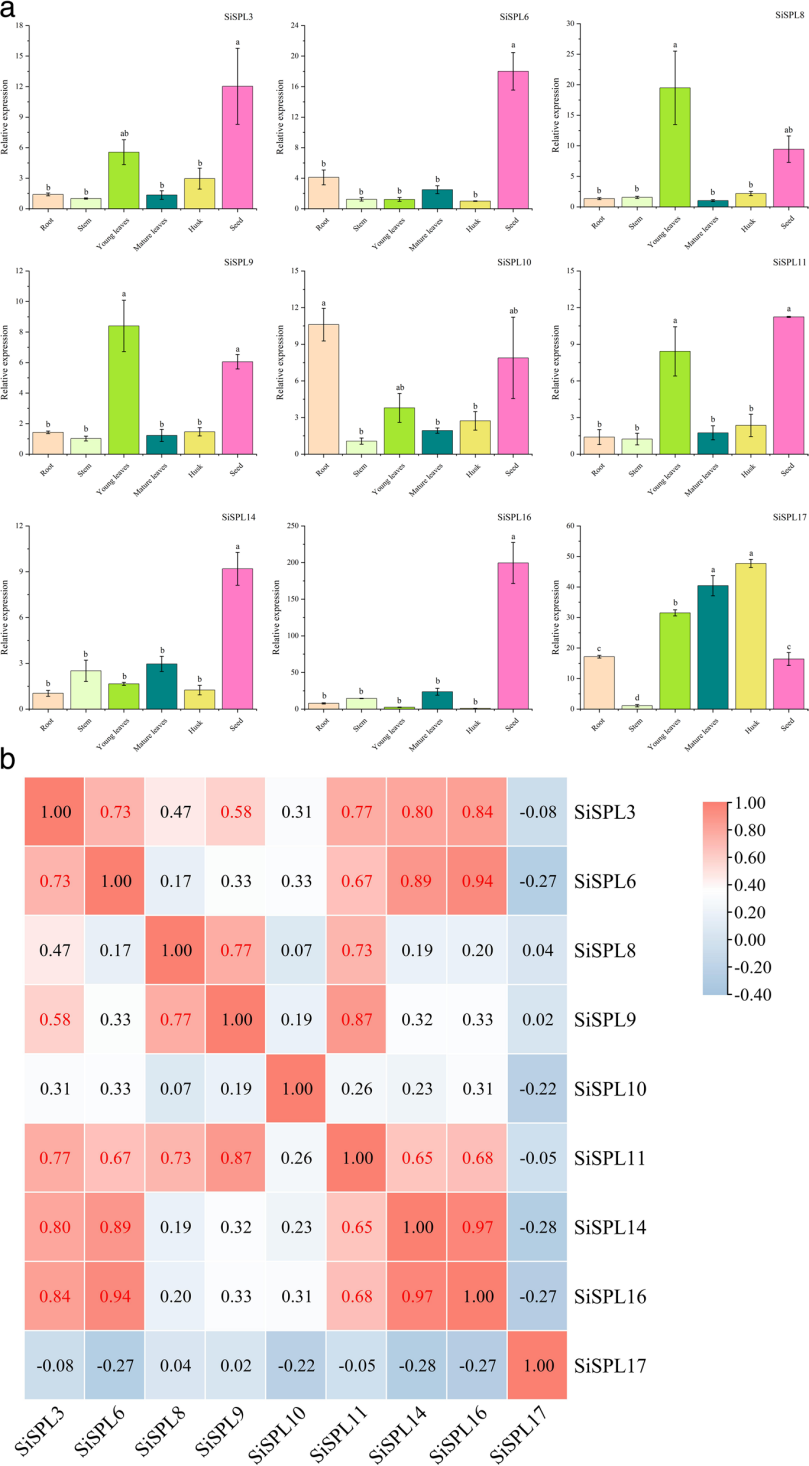
Tissue-specific expression of 9 *S. italica* SPL genes and their correlation with expression patterns in the middle stage of grain filling. **a** Expression patterns of 9 *S. italica* SPL genes in the root, stem, young leaves, mature leaves, husk and seed organs were examined by qRT-PCR. Error bars were obtained from three measurements. Lowercase letter above the bar indicates significant difference (α = 0.05, LSD) among treatments. **b** Positive number: positively correlated; negative number: negatively correlated. Red numbers indicate a significant correlation at the 0.05 level

To better characterize *SPL* gene function in foxtail millet, qRT-PCR was used to determine *SiSPL* gene expression in different tissues (roots, stems, young leaves, mature leaves, husks, and seeds) at the mid-grain filling stage of foxtail millet. We selected at least one representative gene from each subfamily, and nine *SiSPL* genes were detected for their expression patterns in different tissues. As shown in Fig. 8a, different genes have differential expression patterns in different tissues. All genes had extremely high expression levels in seeds (about 10 folds more than controls), and the high expression levels of six genes (*SiSPL3*, *SiSPL8, SiSPL9*, *SiSPL10*, *SiSPL11*, and *SiSPL17*) were observed in young leaves. *SiSPL10* and *SiSPL17* were expressed at higher levels in roots (10 and 15 folds more than the control, respectively). Interestingly, *SiSPL17* also had an extremely high expression profile in husk and mature leaves, unlike the expression profiles of other genes, as reflected by the broader expression abundance of *SiSPL* genes.

In addition, the correlation between gene expression patterns in different tissues was analyzed (Fig. 8b). The results showed that the expression patterns of most genes were positively correlated (*P* < 0.05). For example, three genes, *SiSPL11*, *SiSPL14*, and *SiSPL16*, showed significant positive correlations with each other, and they also showed significant correlations with *SiSPL3* and *SiSPL6*. Interestingly, six genes were negatively correlated with *SiSPL17*, indicating that the expression pattern of *SiSPL17* in tissues was different from that of other genes.

### Expression patterns of *SiSPL* genes in foxtail millet during grain development

**Fig. 9:**
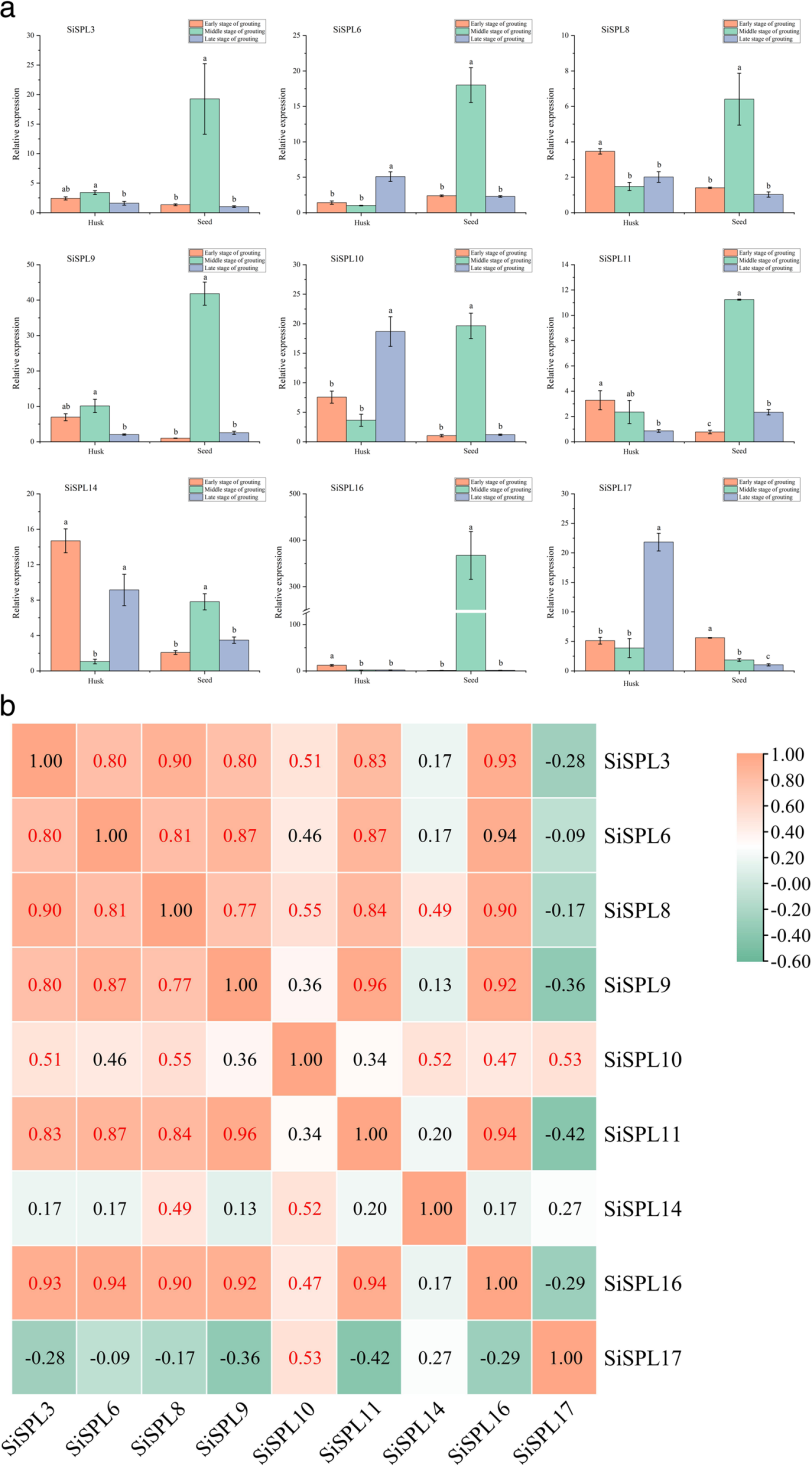
Expression pattern and correlation of 9 *S. italica* SPL genes during grain development. **a** qRT-PCR was used to detect the expression patterns of 9 *S. italica* SPL genes in husk and grain before, during and after grain filling. Error bars were obtained from three measurements. Lowercase letter above the bar indicates significant difference (α = 0.05, LSD) among treatments. **b** Positive number: positively correlated; negative number: negatively correlated. Red numbers indicate a significant correlation at the 0.05 level

The expression of the *SiSPL* gene in different tissues showed that it was mainly highly expressed in seeds. Therefore, we further investigated the expression levels of *SiSPL* in millet grains and husks of early, middle, and late grain filling. As shown in Fig. 9a, the *SiSPL* gene was expressed to varying degrees during seed and husk development at these stages. In addition, *SiSPL17* was highly expressed in the early stage of seed filling and decreased gradually during the filling process, while the other eight genes were highly expressed in the middle stage of seed filling. In addition, genes that had higher expression levels were also observed in the husk during the early stage of filling (*SiSPL8*, *SiSPL10*, *SiSPL11*, and *SiSPL14*) and the later stage of filling (*SiSPL6*, *SiSPL10*, *SiSPL14*, and *SiSPL17*).

We also analyzed the correlation between *SiSPL* gene expression patterns and found that most of these genes were positively correlated (Fig. 9b). For example, significant positive correlations were found between pairs of genes, namely *SiSPL3*, *SiSPL6*, *SiSPL8*, and *SiSPL9*, which were also significantly correlated with *SiSPL11* and *SiSPL16*, respectively. *SiSPL17* was only significantly correlated with *SiSPL10* and positively correlated with *SiSPL11* (P < 0.05), but negatively correlated with all other genes.

### Expression patterns of *SiSPL* genes in response to different abiotic stresses

**Fig. 10:**
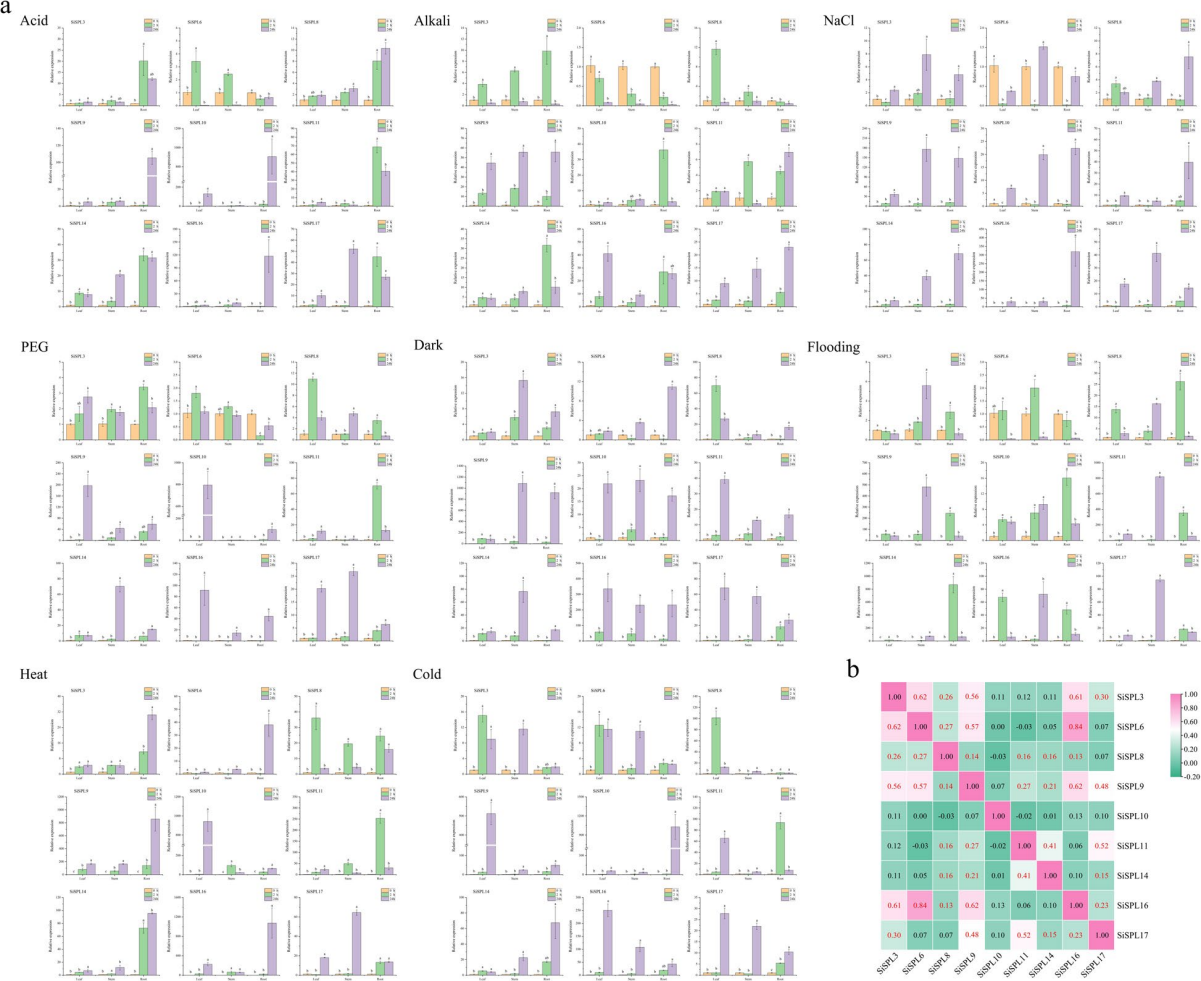
Gene expression of 9 *S. italica* SPL genes in plants subjected to abiotic stresses (acid, alkali, NaCl, PEG, dark, flooding, heat, cold) at the seedling stage. **a** qRT-PCR was used to detect the expression patterns of 9 *S. italica* SPL genes in roots, stems and leaves at different times. Error bars were obtained from three measurements. Lowercase letter above the bar indicates significant difference (α = 0.05, LSD) among treatments. **b** Positive number: positively correlated; negative number: negatively correlated. Red numbers indicate a significant correlation at the 0.05 level

To further determine whether the expression of the *SiSPL* gene was affected by different abiotic stresses, qRT-PCR experiments were carried out on nine SiSPL members to analyze their expression patterns in roots, stems, and leaves under different treatments (acid, alkali, NaCl, PEG, flooding, dark, heat, and cold treatments). In general, some genes were significantly expressed under stress while others were inhibited (Fig. 10a). For example, *SiSPL9*, *SiSPL10*, *SiSPL11*, *SiSPL14*, *SiSPL16*, and *SiSPL17* in roots, stems, and leaves showed different levels of induced expression under all stress conditions, especially *SiSPL9*, *SiSPL10*, and *SiSPL16*(more than 15 folds higher than the control), which showed extremely high levels of induced expression under stress. In contrast, some genes, such as *SiSPL6*, were inhibited under stress, and were down regulated to varying degrees under acid, alkali, NaCl, PEG, and flooding stress. Under stress treatment, more genes tended to reach the highest level of expression at 24 h. For example, under salt stress, the expression of most genes reached a maximum at 24 h.

In addition, a correlation between *SiSPL* gene expression patterns under stress was also observed, and most of the *SiSPL* genes were positively correlated (*P* < 0.05) (Fig. 10b). For example, there was a significant positive correlation between *SiSPL8* and *SiSPL9*, which also showed positive correlations with *SiSPL3*, *SiSPL6*, *SiSPL11*, *SiSPL14* and *SiSPL16*, respectively. A significant positive correlation was found between *SiSPL11* and *SiSPL14*, as well as with *SiSPL17*.

## Discussion

### Characteristics of *SiSPL* genes

The analysis of the physicochemical information of 18 *SiSPL* genes showed that the relative molecular weight of the proteins ranged from 19.2 kDa to 122.18 kDa, with an isoelectric point ranging from 5.58 to 9.91. The following were predicted for subcellular localization: nucleus (16), plastid (1), and chloroplast (1). This result is consistent with the results of *SiSPL* gene domain information alignments, in which a highly conserved nuclear localization signal (NLS) is present in the SBP domain. The analysis of SPL gene families in other species also showed that most *SPL* genes were located in the nucleus [23]. The SBP domain contains highly conserved sequences, such as CQQC, SCR, RRR, NLS, Zn-1, and Zn-2, which is the same as the SBP domain of the SPL gene family in other species [31, 33]. In *AtSPL7* and *OsSPL9* (group IV), the His of the first Zn fingerlike structure was replaced by a Cys residue, which was consistent with *SiSPL8*, suggesting that the Zn fingerlike structure of subfamily I was relatively special. The SPL gene family in foxtail millet can be divided into seven subfamilies, which lack group IV and are different from *Arabidopsis* [18]. The *AtSPL6* gene in group IV plays an important role in plant immunity [50]. In addition, *PpSBP3*, a member of group IV in the bryophyte *Physcomitreiia patens*, can inhibit reproductive development [51]. The results of conserved motif analysis of millet SPL protein showed that foxtail millet *SPL* genes contained the motifs 1 and 2 generally. The conserved motif composition of members of the same subfamily was similar, indicating that the protein structure was conserved in a specific subfamily. However, there are large differences in the conserved motifs among different subfamilies, such as motifs 9, 6, 5, and 10, which are unique to subfamily II (*SiSPL11*, *SiSPL14*, and *SiSPL18*), and none of the other subfamilies contain these four motifs. These motifs may confer special physiological functions to this subfamily, but their specific functions remain to be explored in depth.

The regulation of gene expression by cis-elements in the promoter region has become the main mechanism for organisms to adapt to different environments [52]. Therefore, in addition to studying the structural characteristics of the *SiSPL*s, we also carried out an in-depth analysis of the upstream promoter region of these genes. A large number of cis-acting elements were found in the promoter region. Light response-related elements (G-box, Sp1, and TCCC-motif) were present in most *SiSPL* genes, suggesting that these cis-acting elements may play an important role in the pathway of the *SiSPL*s in plant light response. We also identified root-specific expression elements (as-1) and meristem expression elements (CAT-box) in most of the *SiSPL*s promoter regions. In addition, a large number of *SiSPL* genes have environmental stress-related elements, such as hypoxia-induced (ARE and GC-motif), low-temperature response (LTR), and drought-induced (MBS)-related elements. This also shows that the *SiSPL*s plays an important role in external stress; for example, miR156 in alfalfa improves drought tolerance by silencing *SPL13* [53]. We also found that most *SiSPL*s have cis-acting elements involved in abscisic acid (ABRE), methyl jasmonate (TGACG-motif and CGTCA-motif), and auxin (TGA-element). Methyl jasmonate, a plant hormone and signal molecule associated with injury, exists widely in plants, and its exogenous application can stimulate the expression of defense genes. Previous studies have found that SPL9 interacts with JAZ3 and attenuates the jasmonate (JA) response, making plants resistant to insects in seedlings [54]. At the organ and whole plant levels, auxin acts from seedling to grain ripening. *SPL*s have been found to be involved in the auxin synthesis pathway. Previous studies have shown that *SPL2*, *SPL10*, and *SPL11* can inhibit root regeneration by inhibiting callus-induced auxin synthesis [55]. It was also found that *SPL2*, *SPL10*, and *SPL11* in older plants can directly bind to the promoters of AP2/ERFs to inhibit their expression, thus destroying auxin accumulation in callus [55].

### Evolution of *SiSPL* genes

In higher organisms, introns can regulate gene expression at many levels, and their main role is to produce different exon combinations through alternative splicing to translate different proteins and improve protein complexity [56, 57]. Interestingly, recent studies have shown that introns have important functions independent of the genes they encode. They not only mediates cell responses to starvation [58], but also regulate cell growth rate under stress conditions to improve the adaptability of yeast [59]. The analysis of the *SPL* gene structure in foxtail millet showed that the number of introns in the *SiSPL* gene of groups I and II was much higher than that of other subfamilies. Thus, members of the foxtail millet SPL subfamilies I and II may be older. At present, most studies tend to favor the early intron hypothesis, that is, the existence of a large number of introns in relatively old ancestors, which means that the loss of a large number of introns in evolution is common [60–63]. Research showed that most of the repetitive events of millet genes are generated in whole genome duplication (WGD) events shared by gramineous plants [64]. We investigated the repeat events of the *SiSPL* gene in the whole genome of foxtail millet and found that there were no tandem duplication events; however, four pairs of segmental duplication events existed in the SiSPL gene family. This suggests that segmental duplication events play a major role in the expansion of the *SiSPL* gene over the evolutionary course of the SiSPL gene family. In addition, collinearity analysis with other six species showed that the foxtail millet *SPL* gene had the most homologous genes with maize, followed by rice and sorghum, while tartary buckwheat had the least. Therefore, the millet *SPL* gene has the highest homology with maize. Interestingly, *SiSPL11* has homologous genes with all six species. Thus, we speculated that *SiSPL11* may come from their common ancestor and may have existed before monocotyledon and dicotyledonous differentiation. However, the intron of *SiSPL11* (Fig. 2) also confirmed that this gene may be relatively primitive. Finally, to better understand the evolutionary constraints of the SiSPL gene family, we analyzed the nonsynonymous and synonymous substitutions of the *SiSPL* gene pairs. The results showed that the ka/ks of both the gene pairs of each subfamily and the segmental duplication gene pairs was less than 1, such that the foxtail millet SPL gene family should have experienced strong purification and selection pressure in the process of evolution.

The function of miR156 in regulating plant flowering seems to be conserved, with the overexpression of miR156 in rice [65], *Arabidopsis* [48], maize [66], potato [67], and *Arabis alpina* [68] being associated with a late-flowering phenotype. Among the 17 *SPL* genes in *A. thaliana*, 11 are miR156 targets [18, 28]. In rice, 11 *OsSPL* genes are predictive targets of *Os*miR156 [65]. Among the 18 *SiSPL* genes of foxtail millet, nine members were complementary to miR156. Among them, eight genes were found in the coding region, and one gene (*SiSPL5*) found a potential site that could bind to miR156 in the 3’-UTR region. Interestingly, *AtSPL3*, *AtSPL4*, and *AtSPL5* in group VI also have binding sites for miR156 in the 3’-UTR region, and miR156 can regulate the expression of *AtSPL3* through transcriptional cleavage and translation inhibition to maintain normal plant morphology and flowering time [69]. Therefore, we speculated that *SiSPL5* may be very important for the growth and development of foxtail millet. In addition, *SiSPL* and miR156 complementary site sequence differences were mainly observed in the first, second, and eighth nucleotides, with previous studies reporting similar results [70]. It means that these complementary sites are under great selection pressure in the process of evolution, indicating that miR156-mediated regulation is very important for foxtail millet *SPL* gene expression.

### Spatio-temporal expression patterns of the *SiSPL* gene and its response to abiotic stress

Members of different SPL subfamilies often have different functions, which has been proven in previous studies. Generally, SPL transcription factors are encoded by miR156, but not all *SiSPL* genes have binding sites. For example, *SiSPL8*, a member of subfamily I, does not have binding sites with miR156, which is similar to *AtSPL7*. However, previous studies have shown that *AtSPL7* plays an important role in maintaining copper homeostasis in plants [71]. Similar to the SPL subfamily I genes, the *SPL* genes of subfamily II members are widely expressed in different tissues and at different stages of plant development, but lack the negative regulation of miR156 [22, 31, 72]. *Arabidopsis thaliana* has four SPL subfamily II members, namely *AtSPL1*, *AtSPL12*, *AtSPL14*, and *AtSPL16*, which are widely expressed in seedlings, stem leaves, stem apical meristem, flowers, fruits, and roots [31]. In tomato, the genes *slysbp12a* and *slysbp12b*, two group II members of the SPL subfamily, are widely expressed from seedlings to mature fruits [22]. In this study, *SiSPL11* and *SiSPL14*, members of subfamily II, were also expressed in different tissues (root, stem, leaf, husk, and grain).

The members of rice and *Arabidopsis* SPL subfamily III also lack miR156 binding sites [18, 65], which is consistent with *SiSPL9* (subfamily III). Functional studies of *AtSPL8*, a subfamily III member of *Arabidopsis*, were earlier and clearer. Its genetic mutants greatly affected the seed setting rate, petal trichome production, and root growth, and were found to do so by positively and negatively regulating gibberellin (GA) signaling in flowers and roots, respectively [38, 39]. The analysis of the *SiSPL9* promoter sequence revealed that it contains the participation of a gibberellin response element P-box (CCTTTTG); thus, *SiSPL9* may also be involved in the response pathway to GA signals. In addition, the *SiSPL9* promoter also contains cis-acting elements for hypoxia (ARE and GC-Motif), low temperature (LTR), and drought (MBS), which may result in high levels of *SiSPL9* expression under multiple stress treatments, is supported by the experimental results.

The dicotyledonous Arabidopsis subfamily IV contains only *AtSPL6*, and no homologous gene of *AtSPL6* is found in monocotyledons, indicating that this gene pedigree has been lost in evolution[28]. *AtSPL6* and its homologous gene *NbSPL6* in tobacco play a positive regulatory role in plant innate immunity mediated by the NTIR-NB-LRR receptor [73]. In *A. thaliana*, *AtSPL2*, *AtSPL10*, and *AtSPL11* of SPL subfamily V have binding sites for miR156 [74], and the overexpression of miR156b can reduce the accumulation of *AtSPL2*, *AtSPL10*, and *AtSPL11* mRNA [75]. Functional analysis also showed that *AtSPL2*, *AtSPL10*, and *AtSPL11* can regulate the morphological characteristics of cauline leaves and flowers [76]. Foxtail millet SPL subfamily V contains *SiSPL2*, *SiSPL3*, and *SiSPL10*. *SiSPL3* and *SiSPL10* were expressed in different tissues and periods, and *SiSPL10* was significantly induced under multiple stresses.

The most extensive function of the *SPL* genes are to promote plant growth transition from seedling to mature plant [77, 78]. Overexpression analysis of *AtSPL3*, *AtSPL4*, and *AtSPL5* genes of SPL subfamily VI showed that they are related to changes in plant morphological characteristics [79], and these functions are realized through the negative regulation of miR156 [47, 48, 80]. The SPL subfamily VI in foxtail millet contains *SiSPL5* and *SiSPL6*, and the analysis of the expression pattern of *SiSPL6* showed that it is highly expressed in seeds at the middle filling stage (Fig. 8a) indicating an important role in the adult vegetative growth. In addition, the functions of *AtSPL3*, *AtSPL4*, and *AtSPL5* were verified under different sunshine conditions [81, 82]. Under short-day exposure, miR156 negatively regulates these three genes, and SUPPRESSION OF OVEREX-PRESSION OF CONSTANS1 (SOC1) positively regulates these genes through the GA pathway. Under long-day exposure, SOC1, FLOWERING LOCUS T (FT), and FLOWERING LOCUS D (FD) regulate these three motifs in the leaves to cope with light signals. Both *SiSPL5* and *SiSPL6* have light response elements, G-Box and TCCC motifs, and Sp1, respectively (Additional file Table S3), which suggests that *SiSPL5* and *SiSPL6* may also be involved in light response pathways similar to the *Arabidopsis SPL* subfamily VI genes. Interestingly, the results showed that the expression of *SiSPL6* was not inhibited under dark, cold, or heat stress, but was inhibited to varying degrees under other stress treatments. In addition, *OsSPL13*, a homolog of *SiSPL5* in rice, can positively regulate the cell size in rice husk, thus improving the grain length and yield of rice.

The expression of *SiSPL17* in the same subfamily of *AtSPL13* was highly in roots, leaves, husks, and seeds, except for in the stems (Fig. 8a). *SiSPL12* and *SiSPL15* are found in the same subfamily, which are homologous to *AtSPL13*, *OsSPL2*, and *OsSPL16* (Additional file Table S6). *AtSPL13*, a single member of subfamily VII, was widely expressed in different tissues of *Arabidopsis*, delaying leaf growth after cotyledon emergence during seed germination [83, 84]. *OsSPL16* encodes a protein that positively regulates cell proliferation, and the high expression of this gene can promote cell division and grain filling, thus increasing the grain width and rice yield [44]. In addition, *OsSPL16* directly binds to the *GW7* promoter and inhibits its expression, thereby affecting rice yield and quality [43]. Therefore, the functions of *SiSPL12* and *SiSPL15* in foxtail millet are worthy of further discussion.

In this study, *SiSPL16* was not only highly expressed in different tissues but was also induced under abiotic stress. Subfamily VIII of SPL in foxtail millet includes *SiSPL4*, *SiSPL13*, and *SiSPL16*, in which *SiSPL4* and *SiSPL13* are homologous to upland rice *OsSPL14* and *OsSPL17* (Additional file Table S6). *OsSPL14*, also known as *IPA1*, has been studied extensively and thoroughly, and *OsSPL14* is regulated by miR156 in vivo [41]. *OsSPL14* can inhibit the growth of tiller buds, but promotes the branches of the panicle to increase yield [40, 42, 85, 86], as well as being able to enhance immunity [87]. *OsSPL17* plays an important role in the regulation of rice panicle structure [88, 89]. *AtSPL9* belongs to subfamily VIII of SPL, which participates in petal trichome initiation by activating TRICHOME-LESS (TCL1) and the accumulation of anthocyanin pigments in vegetative stems [90, 91]. In addition, researchers have also found that the miR156-AtSPL9-miR172 regulatory pathway plays an important role in the development of young leaves to mature leaves of *A. thaliana* [92]. The gene function of other members of the subfamily VIII of *Si*SPL warrants further study.

## Materials and methods

### Plant materials, growth conditions, and abiotic stress treatments in foxtail millet

Yugu 1, a cultivar from northern China, was used as the experimental material in this study. And this material was supplied by Prof. Cheng Jianping. For the experimental materials planted in the greenhouse, we obtained the husk and seed in the early, middle, and late filling stages, as well as the roots, stems, and leaves in the middle filling stage. Samples were taken from plants grown under the same conditions (five replicates). The samples were immediately frozen in liquid nitrogen and stored at ‒80℃ for future use. The expression levels of nine *SiSPL* genes from different subfamilies were determined. In addition, foxtail millet plants at seedling stage (28 days) were treated with eight abiotic stresses (salt: 5% NaCl, acid: 0.1 mol/L HCl, alkali: 0.2 mol/L NaOH, drought: 30% PEG6000, flooding: whole plant, darkness: complete shading, heat: 40℃, and cold: 4℃). For acid, alkali, salt and drought stress, the same volume of liquid immersion root system was maintained in different repetitions under the same stress, and the treatment method was referred to Zhang et al. [93, 94]. These genes were sampled for qRT-PCR analysis at 0, 2, and 24 h.

### Total RNA extraction, cDNA reverse transcription, and qRT-PCR analysis

Total RNA was extracted from all samples using an RNA extraction kit (TaKaRa Bio, cat: 9769) and then reverse transcribed (HiScript II Q RT SuperMix for qPCR, cat: R223-01). The qRT-PCR primers for these genes (Additional file Table S9) were designed using Primer 5.0. Si001873 mg (Actin) was used as an internal control. Standard qRT-PCR with SYBR Premix Ex Taq II (TaKaRa Bio, cat: RR820A) was repeated three times on a CFX96 Real-Time System (Bio-Rad). The gene expression was evaluated using the 2^−ΔΔCt^ method.

### Genome-wide Identification of *SiSPL* genes in foxtail millet

We used the *SPL* gene in *Arabidopsis* (https://www.Arabidopsis.org/) and the rice *SPL* gene (http://Rice.plantbiology.msu.edu/index.shtml) to blast foxtail millet genome-wide (https://plants.e Nsembl.org/info/website/ftp/index.html) sequence (score value ≥ 100 and e-value ≤ 1e^−10^), and obtained the candidate foxtail millet *SPL* genes. Then, we downloaded the hidden Markov model (HMM) of SBP domain (PF03110) in Pfam database (http://pfam.xfam.org/) and searched for SPL protein using HMMER 3.0 software (default parameter) (http://HMMER.org/). All candidate millet *SPL* genes were verified using the SMART tool (http://SMART.embl heidelberg.de/). Finally, 18 *SiSPL* genes were obtained, and these genes were analyzed by protein length, MW, PI (https://web.expasy.org/compute_pi/) and protein subcellular localization prediction (https://wolfpsort.hgc.jp/).

### Phylogenetic analysis and classification of the SiSPL gene family

Phylogenetic trees were constructed using protein sequences (*Oryza sativa*, *A. thaliana*, *Solanum lycopersicum*, *Sorghum bicolor*, maize, *and Fagopyrum tataricum*) downloaded from the UniProt Database (https://www.UniProt.org) with muscle wrappers and built an ML phylogenetic tree with IQ-tree wrapper (bootstrap number set to 1000). The classification of the foxtail millet SPL gene family was based on the classification method of the Arabidopsis SPL gene family.

### Chromosomal distribution and gene duplication of *SiSPL* genes

We obtained the physical location information of the *SiSPL* gene from the millet genome and mapped it to chromosomes. The collinearity of *SiSPL* genes was scanned using a Multiple Collinearity Scan toolkit X (MCScanX) with default parameters to analyze gene duplication events. In addition, a dual collinear plotter was used to analyze the homology of *SiSPL* genes between species (https://github.com/CJ-Chen/TBtools).

### Gene structure, conserved motif, cis-acting elements analysis, and prediction of *SiSPL*s targeted by miR156

The coding sequence (CDS) of *SiSPL* was compared with the corresponding genomic DNA sequence to construct a gene structure diagram. The online MEME tool (http://meme-suite.org/tools/meme) was used to analyze the full-length conserved motifs of SiSPL family proteins, and the maximum conserved motif search value was set to 10. We extracted the promoter sequence 2-kb upstream of the *SiSPL* gene and analyzed the cis-acting elements of the promoter region in PlantCARE (http://bioinformatics.psb.ugent.be/webtools/plantcare/html/). We obtained the sequence information of foxtail millet microRNA in the plant microRNA database (PMRD) (http://bioinformatics.cau.edu.cn/PMRD/). After using TAPIR tools (http://bioinformatics.psb.ugent.be/webtools/tapir/) analysis of genomic DNA and cDNA sequences of *SiSPL*s, we predicted the possible targets of miR156. The parameters were set as a score of ≤ 4 and a free energy ratio of ≥ 0.7.

### Statistical analysis

JMP6.0 software (SAS Institute) was used to perform a one-way analysis of variance (ANOVA) of the data, and compared with least significant difference (LSD) at the 0.05 and 0.01 levels. Bar charts were drawn using OriginPro2019b software (OriginLab).

## Supplementary Information

**Additional file 1:** **Table S1.** List of the 18 *Setaria italica*SPL genes identified in this study.

**Additional file 2:** **Table S2.** Analysis and distribution of theconserved motifs in SPL proteins of *Setaria italica* and other species.

**Additional file 3:** **Table S3. ***SiSPL* gene promoter regioncis-acting element details.

**Additional file 4: Table S4. **Information about mir156/172 and *SiSPL*gene binding sites.

**Additional file 5: Table S5. **Segmental duplications of *Setariaitalica* *SPL* genes.

**Additional file 6: Table S6.** Ka/Ks values of each subfamily genepair and all fragment repeat sequence gene pairs.

**Additional file 7: Table S7. **One-to-one orthologous generelationships between *Setaria italica* and other plants.

**Additional file 8: Table S8. **SPL protein sequence information forthe species other than *Setaria italica* in the phylogenetic treeanalysis.

**Additional file 9: Table S9. **Primer sequences for qRT-PCR.

## Data Availability

The entire *Setaria italica* genome sequence information was obtained from the Ensembl Genomes website (http://ensemblgenomes.org/). *S. italica* materials (Yugu 1) used in the experiment were supplied by Prof. Cheng Jianping of Guizhou University. The datasets supporting the conclusions of this study are included in the article and its additional files.
